# Prevalence of mental illness, substance use disorder, and dual diagnosis among adults in custody

**DOI:** 10.1186/s12963-025-00408-7

**Published:** 2025-08-06

**Authors:** Darcy J. Coulter, Lindsay A. Pearce, Matthew Legge, Jesse T. Young, David B. Preen, Ed Heffernan, Jocelyn Jones, Stuart A. Kinner

**Affiliations:** 1 Justice Health Group, enAble Institute, Curtin University, Perth, WA Australia; 2 School of Population Health, Curtin University, Perth, WA Australia; 3 Melbourne Institute: Applied Economic & Social Research, University of Melbourne, Melbourne, VIC Australia; 4 Justice Health Group, Centre for Adolescent Health, Murdoch Children’s Research Institute & Royal Children’s Hospital, Melbourne, VIC Australia; 5 Melbourne School of Population and Global Health, University of Melbourne, Melbourne, VIC Australia; 6 Institute for Mental Health Policy Research, Centre for Addiction and Mental Health, Toronto, ON Canada; 7 Dalla Lana School of Public Health, University of Toronto, Toronto, ON Canada; 8 School of Population and Global Health, The University of Western Australia, Perth, WA Australia; 9 Forensic Mental Health Group, Queensland Centre for Mental Health Research, Brisbane, QLD Australia; 10 Faculty of Medicine, The University of Queensland, Brisbane, QLD Australia; 11 Queensland Forensic Mental Health Service, Queensland Health, Brisbane, QLD Australia; 12 Maladjiny Research Centre, Kurongkurl Katitjin, Edith Cowan University, Perth, WA Australia

**Keywords:** Dual diagnosis, Mental illness, Substance use, Prison, Prevalence, Indigenous, Justice health

## Abstract

**Background:**

The prevalence of mental illness, substance use disorders, and their dual diagnosis is disproportionately high among people in prisons compared to the community. Accurate prevalence estimates are required to inform resourcing of prison health services and reduce the risk of harm to people experiencing these conditions. Existing estimates, where available, often rely on only one data source.

**Method:**

We used three data sources – self-reported history of diagnoses, in-prison medical records, and administrative data to estimate the prevalence of mental illness, substance use disorder, and dual diagnosis among two large cohorts of non-Indigenous and Aboriginal and Torres Strait Islander people in Australian prisons. We calculated population-weighted proportions of the samples with each condition. Inter-rater reliability metrics inform data source agreement.

**Results:**

The prevalence of mental illness only, substance use disorder only, and dual diagnosis was 17.0% (95%CI 12.0–24.5), 14.8% (95%CI 9.6–18.1), and 44.2% (95%CI 33.2–54.7), respectively, for incarcerated, non-Indigenous adults. For incarcerated Aboriginal and Torres Strait Islander adults, our corresponding estimates were 7.0% (95%CI 4.3–11.5), 26.8% (95%CI 18.9–33.5), and 40.9% (95%CI 30.1–48.2). These estimates differed significantly from those derived from singular data sources. Individual data sources’ agreement was weakest for substance use disorder diagnoses and strongest for dual diagnoses.

**Conclusions:**

Individual data sources likely have high specificity and low sensitivity, thus under-ascertaining diagnoses. We recommend using multiple data sources to estimate prevalence to ensure adequate ascertainment of these conditions among people in prison and to ensure in-prison and transitional health services are appropriately resourced.

**Supplementary Information:**

The online version contains supplementary material available at 10.1186/s12963-025-00408-7.

## Introduction

Internationally and in Australia, the prevalence of mental illness, substance use disorder, and their co-occurrence (referred to as dual diagnosis) is higher among people in contact with the criminal justice system than in the general population [1–4]. In a systematic review and meta-analysis of 50 studies, Baranyi et al. [1] reported that the prevalence of co-occurring serious mental illness and substance use disorders among people in prison ranged from 3.5 to 20.7%; these prevalence estimates were between 2 and 20 times higher than community estimates, depending on the type(s) of serious mental illness examined. The factors contributing to disproportionate prevalence of mental illness, substance use disorder, and dual diagnosis in custodial settings are complex but include various social, legal and health factors [5–7]. For people leaving prisons, mental illness, substance use disorders and dual diagnosis increase the risk of negative health outcomes (including premature death) [8, 9], homelessness [10], and reincarceration [11–13] when compared to people leaving prison without these diagnoses. People with dual diagnoses are a particularly vulnerable cohort with higher health needs and more extensive criminal justice system contact than people with mental illness or substance use disorders only [14–17].

Accurate and reliable estimates of mental illness, substance use disorder, and dual diagnosis prevalence in prison settings are required to inform healthcare planning, allocation of resources, healthcare delivery, and appropriate transitional healthcare for those released from prison. Prisons provide a unique setting that removes many of the barriers to healthcare access that incarcerated individuals may have encountered in the community [18]. Unfortunately, healthcare delivered in prisons is often inferior to community healthcare [19, 20] despite the high needs of this population demanding that even equivalent healthcare is not sufficient [21, 22]. Prisons are therefore important settings to identify and treat mental illness and substance use disorders. Despite this, data on health status and outcomes are often not routinely or adequately collected in prison settings [23–26]. Accordingly, accurate estimates of the prevalence of these disorders in prison settings are rare [27]. Further, because people with mental illness, substance use disorder, or dual diagnosis require services and treatments tailored to their specific diagnosis [27, 28], prevalence estimates should distinguish those with singular diagnoses and dual diagnoses.

Despite the importance of accurate and reliable prevalence estimates of mental illness, substance use disorder, and dual diagnosis, many existing estimates suffer from methodological limitations, and comparisons across studies are hindered by methodological differences. To identify previous diagnoses of mental illness and substance use disorder among people in prison, researchers commonly use self-reported data [2, 29–31], screening tools or diagnostic assessments [3, 4, 32–34], administrative data [35, 36], or (to a lesser extent) in-prison medical records [9, 37]. Although each type of data source has both strengths and limitations, very few studies have combined data from multiple sources to estimate the prevalence of mental illness, substance use disorder, and dual diagnosis. Comparisons of prevalence estimates between studies are often complicated by differences in type of data source used, differences in ascertainment period (e.g., 12 months or lifetime), and varying definitions of mental illness, notably whether it includes substance use disorders.

This methodological heterogeneity has contributed to substantial variation in prevalence estimates, even within the same country. For example, in Australia, using diagnostic interviews and screening assessments, Butler et al. [3] estimated the 12-month prevalence of any mental illness among people in prison, inclusive of substance use disorders, to be 80%. Another Australian study using inpatient administrative data for a birth cohort [36] estimated lifetime prevalence of any mental illness, inclusive of substance use disorders, to be 34% among those who had experienced incarceration. In an Australian survey of 371 prison entrants [29] 51% self-reported a lifetime history of any mental illness, inclusive of substance use disorder. Although this marked variation in prevalence estimates may to some extent reflect real variation between settings and cohorts, it is almost certainly in part due to avoidable heterogeneity in ascertainment/measurement methods.

It is unlikely that any singular data source will adequately capture diagnoses to allow for accurate prevalence estimates among people in prisons. Prisons in particular present challenges for accurate ascertainment of mental health conditions due system-level access barriers such as under-resourcing of mental health services [38] and lack of culturally capable staff [39] likely leading to underdiagnosis while incarcerated. Different data sources such as self-report, screening tools, diagnostic tools, medical records, and administrative data each have their own advantages and disadvantages. Self-report can be an efficient way of estimating lifetime prevalence of mental disorder, particularly in circumstances where it is not feasible to obtain diagnostic information from clinical or administrative records. However, self-report is vulnerable to recall bias [40, 41], and studies have demonstrated under-reporting of stigmatised health conditions [42, 43]. Further, surveys of incarcerated populations require significant resources, often resulting in sub-optimal sample sizes. Similarly, screening measures and diagnostic tools are resource-intensive, and as such are rarely used routinely in prison settings. This is particularly the case for diagnostic tools which require highly-trained staff to administer. While screening and diagnostic tools have the advantage of potentially identifying previously undiagnosed conditions, screening tools in particular usually have high sensitivity and low specificity, such that they tend to over-estimate prevalence [27, 44]. Further, information on the cultural validity of many diagnostic measures for Aboriginal and Torres Strait Islander people is unknown [45] meaning the accuracy of diagnoses ascertained through these measures is uncertain. Conversely, prison medical records typically under-estimate lifetime prevalence as current diagnoses are considered more clinically relevant than historical diagnoses. Within Australia (and likely elsewhere), prison medical records will also under-estimate prevalence due to a lack of routine screening for many health conditions, including mental disorder, a lack of culturally competent health services [25, 46, 47], and a reliance on paper-based records which present additional challenges for researchers. Administrative data are collected on whole populations but are limited to the agencies that routinely collect information and may therefore not include many diagnoses occurring in non-acute settings or provided by private practitioners. Administrative data often exclude periods spent in prison which can contribute to under-estimates of prevalence of health conditions for people who have spent time in prison. Under-estimates of lifetime diagnoses may also occur by relying solely on administrative data as they will not capture diagnoses that are outside of the collection timeframe, or diagnoses made in jurisdictions that are not covered by the administrative data source [48].

While using multiple data sources to ascertain health diagnoses is relatively common in other settings [49–51], few studies have estimated the prevalence of mental illness and substance use disorder for people in prison using multiple data sources. We are aware of only two studies that assessed the agreement between self-report and clinical records in the context of anxiety and depression, and alcohol and drug dependence or use in prison settings. Using common interpretations of inter-rater reliability coefficients [52], these studies observed fair agreement across data sources for ever having used illicit drugs [37] and alcohol dependence, moderate agreement for drug dependence, and substantial agreement for anxiety and depression [53]. Neither of these studies statistically compared prevalence estimates.

A further important consideration in estimating prevalence of mental illness and substance use disorders in Australian prisons is the specific health needs of Aboriginal and Torres Strait Islander people. Despite Aboriginal and Torres Strait Islander adults accounting for one third of people in Australian prisons [54], and requiring holistic, culturally-appropriate healthcare services in prison [46, 47, 55–57], prison health services have typically failed to meet their health needs in a culturally capable manner [25, 34, 58]. To ensure that culturally secure and competent healthcare services in prison are adequately funded and resourced for Aboriginal and Torres Strait Islander people, prevalence estimates specific to Aboriginal and Torres Strait Islander people must be available.

In the present study, we aimed to (1) estimate the lifetime prevalence of mental illness, substance use disorder, and dual diagnosis among people in prisons, (2) examine differences in these estimates using different data sources (self-reported history of diagnoses, prison medical records, and linked administrative health data), and (3) quantify the concordance between prevalence estimates generated from these three data sources. We report all results separately for non-Indigenous and Aboriginal and Torres Strait Islander samples.

## Materials and methods

### Study population

We used data from the Health After Release from Prison study, a prospective cohort study of adults recruited prior to release from prisons in Australia. A detailed description of the cohort study methodology is available elsewhere [59]. Due to uncertainty regarding release date, we excluded people held in pre-trial detention (remand). We analysed data from 2,698 adults (18 + years old) incarcerated in Queensland or Western Australia (WA); 1,325 adults recruited from seven prisons in Queensland between August 2008 and July 2010, and 1,373 adults recruited from five prisons in WA between May 2013 and August 2016.

The study aimed to recruit a representative sample of incarcerated adults but we deliberately oversampled females to allow sufficient numbers for sex-stratified analyses for other studies [9]. In Queensland, 21.1% (*n* = 280) of the sample was female and in WA 18.2% (*n* = 250) was female, while the proportion of women in each jurisdiction’s prison system was 7.7% and 9.1%, respectively [60, 61].

Trained research staff carried out eligibility screening, recruitment, and research interviews, independent of correctional authorities. Interviewers read aloud all interview questions, to obviate literacy concerns.

### Baseline measures

Baseline surveys collected information on sociodemographic and criminal justice factors, mental health and substance use, cognitive disability, chronic and infectious disease, and health risk behaviours. Questions regarding lifetime diagnoses of health conditions were adapted from Australia’s National Health Survey [62]. With respect to mental illness, participants were asked: “Have you ever been told by a doctor, psychologist or psychiatrist that you have a mental illness? (No/Yes), and “If Yes, what type of mental illness(es) have you been diagnosed with?” (Anxiety disorder/Depression/Substance abuse/dependence/Schizophrenia/Other [Specify]). We manually recoded responses originally recorded as “Other” to “Substance abuse/dependence” when the further specified information indicated a substance-use diagnosis. Participants were classified as non-Indigenous, or Aboriginal and/or Torres Strait Islander, based on self-report.

### Prison medical records

Prison medical records covering the entirely of the index prison sentence were reviewed and coded by trained researchers using the International Classification of Primary Care - second edition (ICPC-2) [63]. The researchers assigned P70–76, P79–82, P86–98, or P99 codes to the health encounter if a psychologist, psychiatrist or general practitioner had made a diagnosis of mental illness, and P15, P18, or P19 if the health professional had made a diagnosis of substance use disorder.

### Administrative health records

Baseline survey data were retrospectively and prospectively linked to person-level, state-wide hospital and emergency department records. Accredited data integrating authorities in each state conducted probabilistic data linkage using participant name, date of birth, sex, postcode of residence (Queensland only), and all known aliases, using a previously validated method that has been shown to have 99.9% accuracy [64]. Administrative health records included all hospital admissions and emergency department presentations to all state-run public hospitals and licensed private hospitals during the study period within WA and Queensland, respectively. Queensland hospital admission data were available from 1 July 1999 and emergency department data were available from 1 June 2002. Western Australia hospital and emergency department data were extracted for each individual for the five years prior to each participant’s index prison sentence. Western Australia emergency department data were available from January 2002. This resulted in seven individuals (0.5% of the WA sample) in our dataset whose emergency department data did not span the full five-years prior to their incarceration. Variables obtained from inpatient records included primary and secondary diagnosis codes using the International Classification of Diseases, 10th Revision, Australian Modification (ICD-10-AM) [65] and dates of admission and discharge. Variables obtained from emergency department records included primary diagnosis ICD-10-AM code, Major Diagnostic Category (MDC; WA only), and presentation date and time. These ICD-10-AM codes identified hospital admissions and emergency department presentations prior to the baseline survey in which mental illness (F01-09 and F20-99) or a substance use disorder (F10-19) was recorded as a primary or secondary diagnosis. ICD-10-AM codes were missing for 34.8% (3,898) of emergency department encounters in the Western Australian cohort. In these instances, we used MDCs to identify mental illness (MDC 19) and substance use disorder (MCD 20). Major Diagnostic Category is a classification system designed to group patients with similar primary diagnoses together, primarily for billing purposes. Major Diagnostic Categories 19 and 20 encompass the ICD-10-AM codes which we used to define mental illness and substance use disorder, as well as suicidal ideation, hallucinations, unhappiness, and antisocial behaviour (MDC 19); and findings of alcohol and other substances in blood (MDC 20).

### Statistical analyses

#### Ascertainment of diagnoses

We used two slightly different definitions of diagnoses for our prevalence and concordance analyses.

##### Prevalence

For the purposes of estimating lifetime prevalence, we defined dual diagnosis as a diagnosis of both mental illness and substance use disorder in hospital and emergency department data at any point prior to the baseline survey, in prison medical records during the index prison sentence, and/or using baseline survey data. We classified participants into one of four mutually exclusive and collectively exhaustive categories: no mental disorder, mental illness only, substance use disorder only, or dual diagnosis. We constructed four versions of this variable, first using each data source (baseline survey, prison medical records, administrative data) separately, and then combining all available data sources. The variable which used all data sources allowed for dual diagnosis to be coded when we observed a mental illness diagnosis and substance use disorder diagnosis in different data sources for the same individual.

##### Concordance

Agreement analyses used the same methodology as prevalence analyses, but with non-exclusive diagnosis definitions. For these analyses, if an individual had a diagnosis of mental illness and a substance use disorder, we recorded that person as having a mental illness diagnosis, a substance use disorder diagnosis, and dual diagnosis. This differs from the exclusive mental illness *only* and substance use *only* definitions used in our prevalence analyses.

#### Main analyses

We generated prevalence estimates by calculating the proportion of the sample with each diagnosis, while also incorporating probability sample weights with bootstrapping to account for non-random sampling of participants. Sample weights were created using prisoner population statistics on age, sex, Indigenous status, and number of sentenced prisoners at 30 June 2010 for Queensland participants [66] and 2016 for Western Australia participants [67]. We conducted paired-samples z-tests to identify differences in prevalence estimates between individual data sources and all three data sources combined. We did not further stratify estimates by sex due to the relatively small number of females.

We conducted analyses to assess agreement of diagnoses at the individual level, given that similar prevalence estimates in each data source do not necessarily indicate good agreement. To illustrate the overlap in diagnosis ascertainment across data sources, we created Venn diagrams using the eulerr package [68] in R (v.4.3.2) [69]. To measure agreement across data sources at the individual level, we calculated two inter-rater reliability statistics; prevalence and bias-adjusted kappa (PABAK) [70, 71], and Gwet’s agreement coefficient (AC) [72]. These metrics are appropriate in situations where prevalence is not close to 50% [73]. We interpreted our results using probabilistic benchmarking [74] applied to common guidelines [52].

We conducted all analyses separately for non-Indigenous and Aboriginal and Torres Strait Islander sub-samples. Analysing these sub-samples separately and refraining from direct comparisons between the sub-samples allows for discussion of specific health needs for each cohort and aligns with best practice [75]. We ran all analyses (apart from the Venn diagrams) using STATA version 18 [76].

#### Sensitivity analyses

To assess how our choice of left-censoring date, the proximity of diagnoses, and the use of MDCs affected prevalence estimates, we conducted sensitivity analyses using the linked administrative data only. First, we restricted the data to diagnoses recorded no more than five years prior to the baseline survey. Second, we applied a more restrictive definition of dual diagnosis that mental illness and substance use disorder diagnoses to have occurred within 12 months of each other. In a third sensitivity analysis we removed participants whose diagnoses relied solely on MDCs and compared prevalence estimates from this restricted sample to the full sample.

### Ethical review

The study received ethics approval from The University of Queensland’s Behavioural and Social Sciences Ethical Review Committee (2007000607). Approval for linkage to the Emergency Department Information System and the Queensland Hospital Admitted Patient Data Collection was provided by the Queensland Government Department of Health (HREC/11/QHC/40) under the Queensland Public Health Act (2005) [77] (RD004706). The Western Australian arm of the study was also approved by the University of Western Australia Human Research Ethics Committee (RA/4/1/5076). Approval for linkage to the WA Emergency Department Data Collection and Hospital Morbidity Data Collection was provided by the Western Australia Department of Health (RGS0000000110). Approvals to access prison medical records were provided by Queensland Corrective Services and the WA Department of Corrective Services, Research Application Assessment Committee (Approval no. 306). All participants provided written, informed consent to participate in baseline surveys. Participants in WA also gave consent for linkage with state health records; data linkage in Queensland was undertaken with a waiver of consent approved under the Queensland Public Health Act (2005).

## Results

### Descriptive analysis

Baseline characteristics for the non-Indigenous and Aboriginal and Torres Strait Islander samples are presented in Table 1. After excluding participants who did not have their administrative health records linked, we retained 2,645 (98.0%) of the 2,698 participants for analysis. A total of 1,667 participants (63.0%) were non-Indigenous and 978 (37.0%) identified as Aboriginal and/or Torres Strait Islander. The non-Indigenous sample was predominantly male (*n* = 1392, 83.5%) and more than half were incarcerated in Queensland (*n* = 979, 58.7%). The Aboriginal and Torres Strait Islander sample was predominantly male (*n* = 732, 74.9%) and around two thirds were incarcerated in Western Australia (*n* = 642, 65.6%).

**Table 1:** Self-reported baseline characteristics of the cohort

	Self-reported Indigenous status			
	Non-Indigenous (n= 1667)		Aboriginal and Torres Strait Islander (n = 978)	
	n	%	n	%
Sex				
Male	1392	83.5	732	74.8
Female	275	16.5	246	25.2
Age in years				
< 25	465	27.9	334	34.2
25–39	838	50.3	542	55.4
40+	364	21.8	102	10.4
State				
Queensland	979	58.7	336	34.4
WA	688	41.3	642	65.6
Sexual identity				
Heterosexual	1594	95.6	920	94.1
Gay/Lesbian	30	1.8	18	1.8
Bisexual	42	2.5	38	3.9
Stable relationship at baseline	640	38.4	378	38.7
Number of school years completed^a^				
< 10	555	33.4	501	51.8
>=10	1105	66.6	467	48.2
Ever injected drugs	931	55.8	576	58.9
Ever overdosed	428	25.7	198	20.2
Self-rated health^b^				
Excellent	150	21.9	187	29.4
Very good/good	467	68.2	397	62.5
Fair/poor	68	9.9	51	8.0
Ever self-harmed	97	5.8	118	12.1
Under influence of alcohol and/or drugs at time of offence^c^	1130	67.9	752	76.9

WA = Western Australia

^a^*n*_Non-Indigenous_=1660, *n*_Aboriginal Torres Strait Islander_ =968

^b^ *n*_Non-Indigenous_=685, *n*_Aboriginal Torres Strait Islander_ =635

^c^ *n*_Non-Indigenous_=1665, *n*_Aboriginal TorresStrait Islander_=978

### Prevalence of diagnoses by data source

#### Non-Indigenous sample

**Fig. 1:**
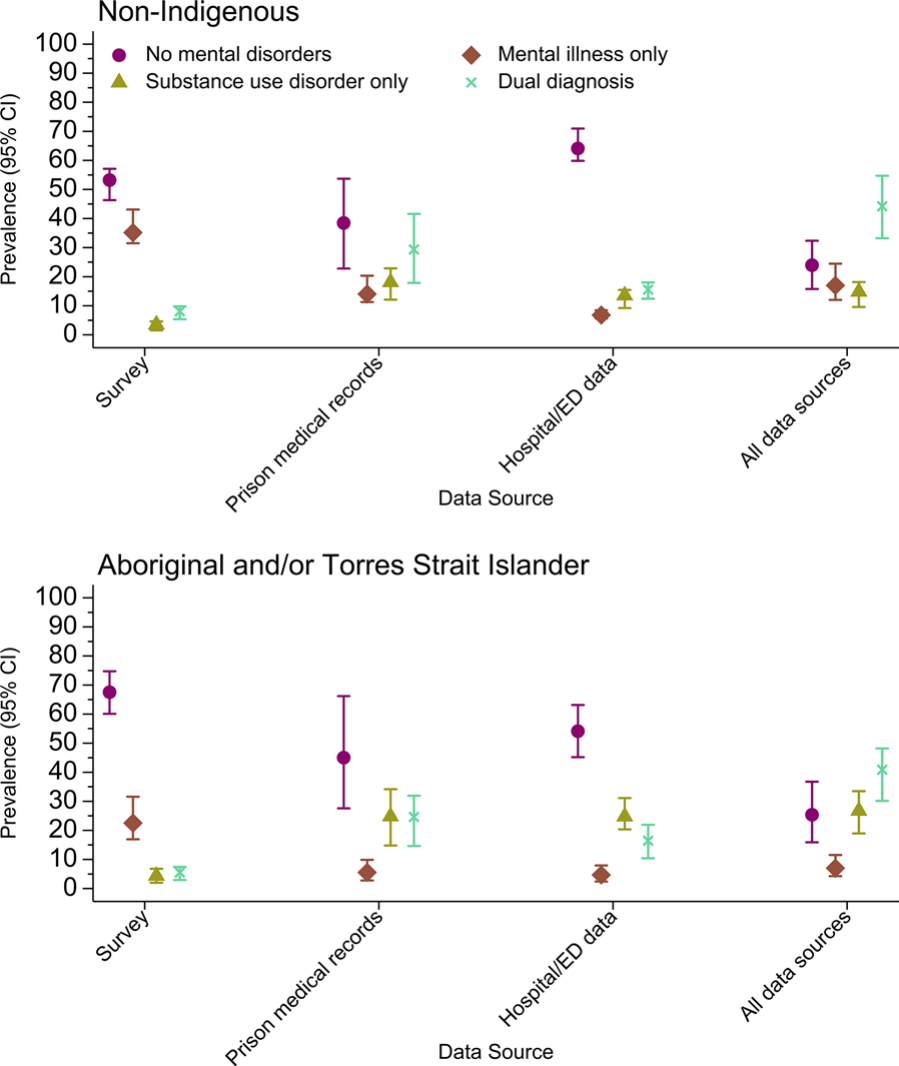
Prevalence of mental illness, substance use disorder, and dual diagnosis using multiple data sources for Non-Indigenous and Aboriginal and Torres Strait Islander samples

Table 2 Fig. 1 present the prevalence estimates of no diagnosis, mental illness only, substance use disorder only, and dual diagnosis for both cohorts. By using information from all three data sources, we estimated the prevalence of mental illness only, substance use disorder only, and dual diagnosis among non-Indigenous people to be 17.0% (95%CI 12.0–24.5), 14.8% (95%CI 9.6–18.1), and 44.2% (95%CI 33.2–54.7), respectively. Each individual data source had lower estimated prevalence for dual diagnoses, when compared to all three data sources combined. Specifically, for dual diagnosis estimates, and using the combined data sources, we demonstrated higher prevalence estimates than the survey (8.1%, 95%CI [5.4, 9.8], *z* = −6.8, *p* <.001), prison medical records (29.4%, 95%CI [17.8, 41.6], *z* = −6.8, *p* <.001), or linked administrative data (15.5%, 95%CI [12.4, 18.0], *z* = −6.0, *p* <.001) individually. Prevalence estimates of substance use disorder in the survey data alone (3.5%, 95% CI [1.5, 4.6], *z* = −6.2, *p* <.001) were lower than the combined data sources (14.8%, 95% CI [9.6, 18.1]). Using the combined data sources did not always produce higher prevalence estimates when examining mental illness only and substance use disorder only though. This was an artefact of our mutually exclusive diagnosis categories, and we therefore limit our discussion of these differences.

**Table 2:** Prevalence of mental illness, substance use disorder, and dual diagnosis using multiple data sources for aboriginal and Torres Strait Islander and Non-Indigenous samples

	Survey			Prison medical records			Hospital/ED			All data sources	
	n	% [95%CI]	z-value *(p)*	n	% [95%CI]	z-value *(p)*	n	% [95%CI]	z-value *(p)*	n	% [95%CI]
**Non-indigenous (n = 1667)**											
No diagnosis	895	53.2 [46.3, 57.1]	6.1 (<.001)	655	38.5 [22.8, 53.7]	3.1 (.002)	1054	64.1 [59.8, 71.0]	11.3 (<.001)	399	24.0 [15.8, 32.4]
Mental illness only	579	35.2 [31.5, 43.1]	5.9 (<.001)	231	14.0 [11.3, 20.3]	−1.4 (.165)	119	6.8 [5.6, 8.4]	−2.8 (.004)	287	17.0 [12.0, 24.5]
Substance use disorder only	63	3.5 [1.5, 4.6]	−6.2 (<.001)	292	18.1 [12.1, 22.8]	2.4 (.017)	233	13.5 [9.2, 15.4]	−1.0 (.336)	259	14.8 [9.6, 18.1]
Dual diagnosis	130	8.1 [5.4, 9.8]	−6.8 (<.001)	489	29.4 [17.8, 41.6]	−6.8 (<.001)	261	15.5 [12.4, 18.0]	−6.0 (<.001)	722	44.2 [33.2, 54.7]
**Aboriginal and Torres Strait Islander (n = 978)**											
No diagnosis	638	67.6 [60.1, 74.7]	6.8 (<.001)	307	45.0 [27.6, 66.2]	3.3 (.001)	480	54.1 [45.2, 63.2]	6.4 (<.001)	166	25.4 [15.9, 36.8]
Mental illness only	245	22.5 [16.9, 31.6]	6.3 (<.001)	83	5.5 [2.8, 9.9]	−0.8 (.444)	55	4.7 [2.4, 7.9]	−1.4 (.164)	66	7.0 [4.3, 11.5]
Substance use disorder only	39	4.4 [2.0, 6.8]	−5.2 (<.001)	295	24.8 [14.8, 34.2]	−0.3 (.733)	234	24.8 [20.4, 31.1]	−0.7 (.461)	281	26.8 [18.9, 33.5]
Dual diagnosis	56	5.5 [3.0, 7.4]	−8.1 (<.001)	293	24.6 [14.6, 31.9]	−6.9 (<.001)	209	16.4 [10.4, 21.9]	−9.6 (<.001)	465	40.9 [30.1, 48.2]

Survey = Self-report of ever being diagnosed by a medical professional. Hospital/ED = Statewide hospital and emergency department linked administrative data. % = Bootstrapped prevalence estimates (10,000 repetitions) of subsample diagnosed using each data source and sample weighted by Australian prisoner population statistics, represented by percentage. CI = Bootstrapped bias-corrected confidence intervals. z-value = Z test comparing prevalence estimate from individual data source to prevalence estimate obtained using all data sources combined

As expected, both our two restrictions on timing of diagnoses ascertained through the linked administrative data resulted in lower dual diagnosis prevalence estimates (5-year pre-survey window: 11.9%, 95%CI [8.6, 14.5], *z* = −6.6, *p* <.001; 12-month co-occurrence: 14.0%, 95%CI [11.1, 16.0], *z* = −5.2, *p* <.001) than the unrestricted linked administrative data analyses (15.5%, 95%CI [12.4, 18.0]). Removing diagnoses ascertained through MDCs did not result in significantly different estimates than the unrestricted analyses. Table S1 presents these results.

#### Aboriginal and Torres Strait Islander sample

For Aboriginal and Torres Strait Islander people in Queensland and WA prisons, using all three data sources, we estimated the prevalence of mental illness only, substance use disorder only, and dual diagnosis to be 7.0% (95%CI 4.3–11.5), 26.8% (95%CI 18.9–33.5), and 40.9% (95%CI 30.1–48.2), respectively. Estimating prevalence using singular data sources resulted in higher or lower estimates than the combined data sources given we coded the diagnoses to be mutually exclusive. Specifically, for dual diagnosis, and using the combined data sources, we produced higher prevalence estimates (40.9%, 95%CI [30.1, 48.2]) than the survey (5.5%, 95%CI [3.0, 7.4], *z* = −8.1, *p* <.001), prison medical records (24.6%, 95%CI [14.6, 31.9], *z* = −6.9, *p* <.001), and linked administrative data (16.4%, 95%CI [10.4, 21.9], *z* = −9.6, *p* <.001) individually. We estimated lower prevalence for substance use disorder only using survey responses only (4.4%, 95% CI [2.0, 6.8], *z* = −5.2, *p* <.001), when compared to all data sources combined (26.8%, 95% CI [18.9, 33.5]). Again, our mutually exclusive coding of diagnoses explained instances where the combined data sources produced lower prevalence estimates of mental illness only than those estimated using individual data sources.

Sensitivity analyses suggested no significant differences in prevalence estimates obtained through the inpatient and emergency department data when we restricted ascertainment of dual diagnosis to individual diagnoses occurring within the same 12-month period, compared to the unrestricted administrative data. When we restricted ascertainment of diagnoses to be within the five years prior to baseline survey, dual diagnosis estimates (12.6%, 95%CI [7.9, 16.8], *z* = −4.2, *p* <.001) were lower than the unrestricted data (16.4%, 95%CI [10.4, 21.9]). Removing individuals whose diagnoses relied on MDCs did not produce statistically significantly different prevalence estimates for any of the diagnoses.

### Data source agreement

#### Non-Indigenous sample

**Fig. 2:**
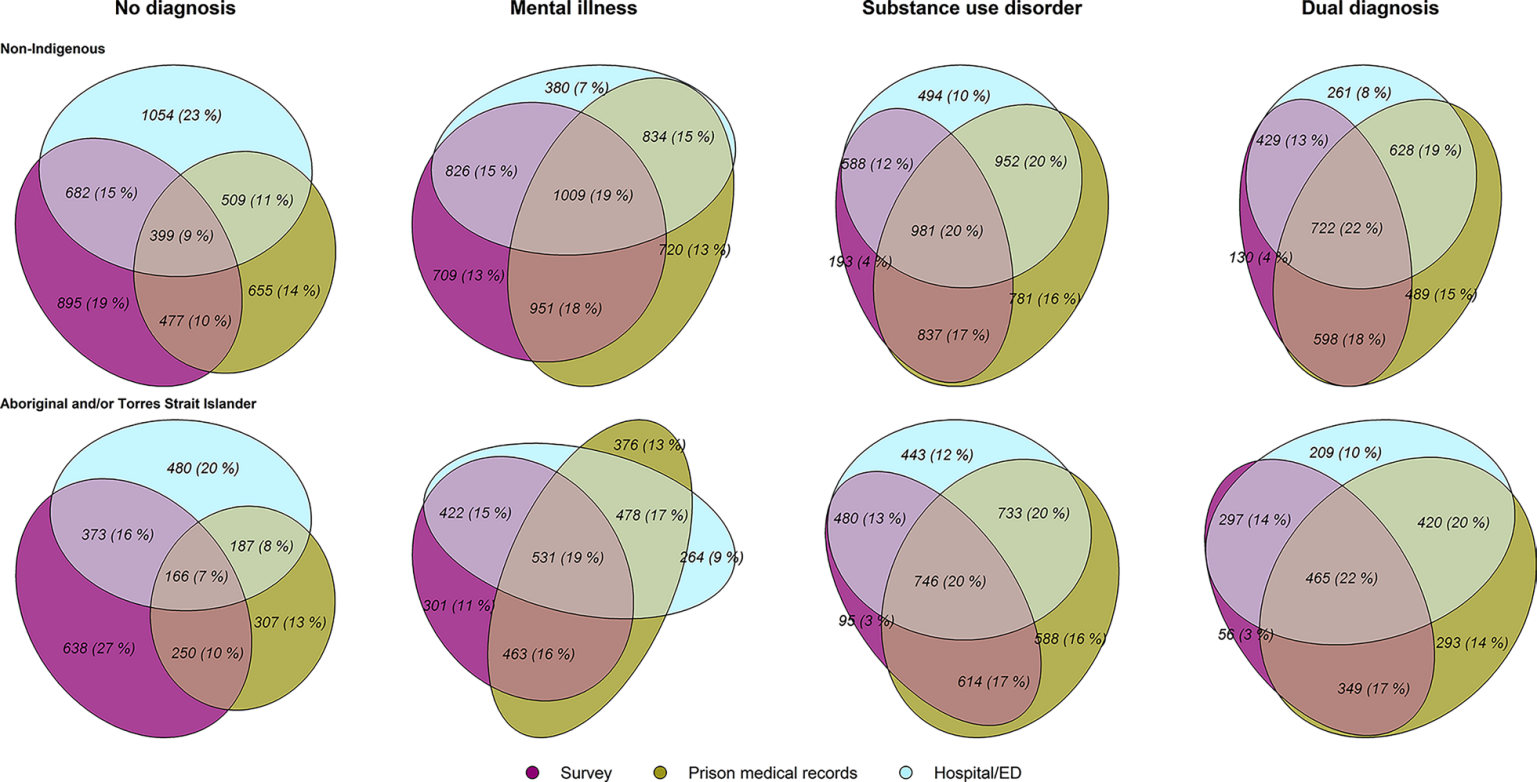
Overlap of data sources for diagnoses of mental illness, substance use disorder, and dual diagnosis: Non-Indigenous sample (*n* = 1,667), Aboriginal and Torres Strait Islander sample (*n* = 978)

Figure 2 demonstrates the degree of overlap in diagnoses across all data sources. For people who received a diagnosis of mental illness, substance use disorder, or dual diagnosis, all three data sources agreed on these diagnoses for only 19%, 20%, and 22% of individuals, respectively. For diagnoses involving substance use disorder (substance use disorder and dual diagnosis), we ascertained 4% through survey results only. We ascertained 13% of mental illness diagnoses through self-reports alone. Inter-rater reliability statistics demonstrated fair agreement among data sources for mental illness (PABAK = 0.36, AC = 0.40), substance use disorder (PABAK = 0.27, AC = 0.38), and moderate (PABAK = 0.52) to substantial (AC = 0.66) agreement for dual diagnosis. Table 3 presents the full inter-rater reliability results.

**Table 3:** Agreement between survey, prison medical records, and combined hospital and emergency department sources for mental illness, substance use disorder, and dual diagnoses with aboriginal and Torres Strait Islander and Non-Indigenous samples

	Gwet’s AC	PABAK
**Non-Indigenous (n = 1667)**		
No mental illness	.25	.25
Mental illness	.40	.36
Substance use disorder	.38	.27
Dual diagnosis	.66	.52
**Aboriginal and Torres Strait Islander (n = 978)**		
No mental illness	.16	.16
Mental illness	.49	.42
Substance use disorder	.09	.04
Dual diagnosis	.61	.46

Gwet’s AC = agreement coefficient [72]. PABAK = Prevalence and bias-adjusted kappa [70, 71]

#### Aboriginal and Torres Strait Islander sample

The level of overlap in diagnoses across data sources was also low for Aboriginal and Torres Strait Islander participants. For participants who received a diagnosis of mental illness, substance use disorder, or dual diagnosis, all three data sources agreed on these diagnoses for 19%, 20%, and 22% of individuals, respectively (Fig. 2). We ascertained a small percentage of substance use disorder diagnoses and dual diagnoses (3% each) through survey results alone. Comparatively, we ascertained 11% of mental illness diagnoses through self-reports alone. Inter-rater reliability tests suggested fair (PABAK = 0.42) to moderate (AC = 0.49) agreement across the data sources for mental illness. Data sources only slightly agreed for substance use disorder diagnoses (PABAK = 0.04, AC = 0.09) and moderately agreed for dual diagnosis (PABAK = 0.46, AC = 0.61).

## Discussion

Using innovative data triangulation, the present study produced prevalence estimates of mental illness, substance use disorders, and their dual diagnosis among Aboriginal and Torres Strait Islander and non-Indigenous people in prison. Given our enhanced ascertainment methods, our estimates are likely closer to the true prevalence of these diagnoses than previously estimated among people in prison. Our results suggest that over 40% of people in Australian prisons have been diagnosed with both a mental illness and substance use disorder. This is substantially higher than our estimates for those who have not received diagnoses or who have received a diagnosis of either mental illness or a substance use disorder, but not both. The study also demonstrates that the prevalence of mental illness, substance use disorders, and dual diagnosis is highly dependent on the source(s) of data used. Due to the likely low sensitivity but high specificity of each data source, our findings show that using multiple data sources provides greater capture of diagnoses and likely more accurate estimates of prevalence of these conditions.

Our datasets are presumably characterised by low sensitivity and high specificity, meaning diagnoses we do capture are likely true positives, but we also expect to encounter many false and true negatives. Characteristics of our datasets such as diagnoses recorded by medical professionals (in-prison medical records and administrative data) and no foreseeable advantage to disclosing non-existent diagnoses to the interviewer (self-report) likely result in data sources of high specificity. Restricted timeframes on diagnoses and only clinically relevant and presenting problems being recorded (in-prison medical records and administrative data), and the likely under-reporting of stigmatised health conditions (self-report) likely resulted in low specificity. Combining data sources of this type may reduce the number of false negatives, thus improving sensitivity. We ascertained more total diagnoses after combining data but given that our diagnosis categories were mutually exclusive, the direction of change in ascertainment for each outcome separately was not always positive.

Using data triangulation, we estimated dual diagnosis prevalence to be 44.2% for the non-Indigenous sample, and 40.9% for the Aboriginal and Torres Strait Islander sample. While study variations such as differences in jurisdictions, type of data source used, differences in ascertainment period (e.g., 12 months or lifetime), and varying definitions of mental illness, make comparisons of prevalence estimates across studies difficult, we observed higher dual diagnosis prevalence than in a previous similar study. Lifetime prevalence of dual diagnosis for people in prison in NSW was estimated (through self-report) to be 23.7% (males) and 26.5% (females) in 2009 and 18.1% (males) and 29.6% (females) in 2015 [2]. Our use of multiple data sources likely contributed to the higher estimates in the present study.

By reporting mutually exclusive prevalence estimates, we highlight the overlap of mental illness and substance use disorders among people in prison. For example, while Browne et al. [2] reported lower dual diagnosis estimates than in our study, they estimated prevalence of lifetime mental illness diagnosis (45.0% [2009], 63.1% [2015]) to be higher than our self-report estimates (non-Indigenous = 35.2%, Aboriginal and Torres Strait Islander = 22.5%). This difference was greater when we used multiple data sources (non-Indigenous = 17.0%, Aboriginal and Torres Strait Islander = 7.0%) estimates. Accurate estimates of these mutually exclusive diagnoses are important to inform the resourcing needs for dual diagnosis-specific services (e.g., integrated mental health and alcohol and other drug services) and services required for mental illness and substance use disorders only. While the integration of these services may be costly, we have demonstrated that a large proportion of the population in prison likely require these services. A previous study using the Queensland cohort demonstrated 3.5-fold higher healthcare costs and 2.8-fold higher reincarceration costs for people with dual diagnosis compared to those with no diagnosis, mental illness only, or substance use disorder only diagnoses [78]. This may mean that the costs to establish tailored dual diagnosis services could be offset by savings in reduced acute presentations and reincarcerations. We have minimised any discussion of differences between individual data sources for mental illness only and substance use disorder only prevalence estimates as these differences may partly be an artefact of using mutually exclusive categories.

Despite this, findings regarding substance use disorder diagnoses highlight the risk of underestimating prevalence when relying on a single data source. The number of individuals identified as having a substance use disorder based on self-report was notably lower than the number identified through either prison medical records or administrative data. Under-reporting of substance use is common, as disclosure may result in real or perceived consequences including shame, discrimination, reinforcement of stigma, implications for release from prison, health care, housing, employment, and child protection system involvement [42, 43, 79–83]. These consequences are especially relevant for people soon to be released from prison given this cohort’s high rates of contact with justice and child protection systems [84] and the existing barriers they face to secure employment [85] and housing [86]. Non-disclosure of substance use disorder diagnoses may also not be a deliberate attempt to conceal use. Recall accuracy of diagnoses received in acute settings such as emergency departments has been shown to be low, with misunderstandings, inadequate communication, and unawareness of diagnoses suggested as explanations [40, 87, 88]. Given potential under-reporting of conditions, data sharing across sectors (e.g. corrections and health), and between community and prison settings is crucial for quality continuity of care [89]. While structural limitations and resistance from governments/prison operators may hinder this data sharing, its importance is recognised by the World Health Organisation’s “whole-of-government approach to healthcare in prisons, which to be effective requires data sharing [26, 90].

Consistent with principles of Indigenous data sovereignty [75], in this study we conducted our analyses separately for Aboriginal and Torres Strait Islander and non-Indigenous people and refrained from direct comparisons of their prevalence. Nevertheless, our findings with regard to both prevalence estimates and concordance analyses were similar in both groups. One notable exception to this is that for Aboriginal and Torres Strait Islander people, prevalence estimates for mental illness only and substance use disorder only were similar between the combined data sources and prison medical records and administrative data, whereas this was not always the case for the non-Indigenous sample. This suggests there may be systematic differences in the diagnoses captured by data sources for each cohort. Overall, our findings suggest that combining data sources likely improves ascertainment of mental illness and substance use disorder for both Aboriginal and Torres Strait Islander and non-Indigenous people in custodial settings.

### Strengths and limitations

To our knowledge, this is the first study to examine the ascertainment of mental illness, substance use disorder, and dual diagnosis in a prison setting, using multiple data sources. This use of a novel data triangulation method for ascertaining mental and behavioural disorders is valuable in this context given the high prevalence of mental illness, substance use disorder, and dual diagnosis in prison settings; the importance of prison settings for identifying dual diagnosis among a population that often has low rates of health care access in the community; and the need for reliable prevalence estimates to inform the design and resourcing of prison health care services.

Our study has four main limitations. First, we focussed only on known diagnoses. Given the barriers to healthcare in the community that this population often faces [91, 92], the proportion of undetected diagnoses may be higher among this cohort than the general community. Though apart from self-report, the only community healthcare encounters our study captured were acute hospital presentations where these barriers may be less likely to exist. This is our second limitation. Although we were able to capture diagnoses through self-report, prison medical records, and emergency department and hospital data, we did not have access to other data that may have recorded diagnoses, such as community mental health and alcohol and other drug treatment services. As such, our prevalence estimates, although high, are likely conservative. Third, while the use of multiple data sources likely improves ascertainment of mental and behavioural disorders, without a ‘gold standard’ (e.g., diagnostic interview with a culturally capable psychiatrist [32]) to compare against we cannot be certain that our prevalence estimates are accurate. Fourth, our study relies on diagnoses derived from Western-centric medical and health systems. Given the holistic nature of social and emotional wellbeing, and importance of connection to country and community for many Aboriginal and Torres Strait Islander people [93, 94], the diagnoses we relied on may not be as informative for the treatment needs for this sample as they are for the non-Indigenous sample.

## Conclusions

People in contact with criminal justice systems experience disproportionate health burden, including high rates of mental illness, substance use disorder, and dual diagnosis. Given that these mental and behavioural disorders are associated with adverse health and justice outcomes, and that people leaving prisons face many barriers to healthcare access in the community, prisons are an important setting to identify and commence treatment for these disorders. To ensure that mental health and substance use services are adequately resourced and available, it is necessary to have accurate prevalence estimates. To ensure that appropriate services in prison are offered to those who require them, high case detection by prison health services is required. However, health data in prisons are often limited and existing estimates and screening rely heavily on a single data source, often self-report.

Our findings indicate that a substantial proportion of people in Australian prisons have dual diagnoses, and that prevalence estimates are highly dependent on the data source used to identify diagnoses. Therefore, this study supports the use of multiple data sources to ascertain mental illness, substance use disorder, and dual diagnosis in prison settings. We suggest that using multiple data sources to estimate prevalence of known mental illness and substance use disorder diagnoses, as well as their co-occurrence, may protect against the limitations and biases of each individual data source. Accurate ascertainment is needed to adequately fund and resource in-prison and transitional mental health and substance use services. Use of multiple data sources will also increase the capacity of prison health services to identify, and offer appropriate treatment to, people entering custody with these conditions and improve release planning in terms of health needs.

## Supplementary Information

Supplementary Material 1

## Data Availability

Data cannot be shared publicly because of ethical requirements and the sensitive nature of these data. Data are available subject to approval from all relevant data custodians and ethics committees. The baseline survey data were collected by some of the authors and are subject to multiple ethics and research committees’ approvals. The administrativedata include sensitive personal information that has been obtained with a waiver of consent, with approval from the relevant data custodians. These data are owned by government agencies and the approval of these agencies is required for access to the data to be granted.
