# Supplementary material for: Prevalence of mental illness, substance use disorder, and dual diagnosis among adults in custody
Source: Popul Health Metr. 2025 Aug 6;23:45. doi: 10.1186/s12963-025-00408-7 (PMC12326852; doi:10.1186/s12963-025-00408-7)
Supplement: Supplementary file 1 — Supplementary Material 1 [file 12963_2025_408_MOESM1_ESM.docx]

| **Supplementary Materials**  Table S1. Prevalence of mental illness, substance use disorder, and dual diagnosis using emergency department and inpatient admission data and various restrictions for Aboriginal and Torres Strait Islander and Non-Indigenous samples | | | | | | | | | | | | | | | |
| --- | --- | --- | --- | --- | --- | --- | --- | --- | --- | --- | --- | --- | --- | --- | --- |
|  | 5-year restriction ^a^ | | |  | Within 12-month restriction ^b^ | | |  | MDC removed ^c^ | | |  | | Unrestricted ^d^ | |
|  | n | %  [95%CI] | z-value  (*p*) |  | n | %  [95%CI] | z-value  (*p*) |  | n | %  [95%CI] | z-value  (*p*) |  | | n | %  [95%CI] |
| **Non-Indigenous (n=1667)** | | | | | | | | | | | | | | | |
| No diagnosis | 1166 | 72.0  [67.7, 77.8] | 11.5  (<.001) |  | 1065 | 64.7  [60.3, 71.2] | 1.6  (.116) |  | 1053 | 64.7  [60.3, 71.4] | 2.0  (.046) | | 1054 | | 64.1  [59.8, 71.0] |
| MI only | 103 | 5.3  [4.3, 6.5] | -2.6  (.010) |  | 116 | 6.8  [5.4, 8.4] | -0.1  (.959) |  | 114 | 6.5  [5.3, 8.4] | -0.7  (.459) | | 119 | | 6.8  [5.6, 8.4] |
| SUD only | 193 | 10.8  [7.8, 12.4] | -3.8  (<.001) |  | 256 | 14.6  [10.6, 16.9] | 2.2  (.028) |  | 229 | 13.4  [9.1, 15.3] | -1.6  (.113) | | 233 | | 13.5  [9.2, 15.4] |
| Dual diagnosis | 205 | 11.9  [8.6, 14.5] | -6.6  (<.001) |  | 230 | 14.0  [11.1, 16.0] | -5.2  (<.001) |  | 255 | 15.3  [12.4, 17.7] | -1.4  (.165) | | 261 | | 15.5  [12.4, 18.0] |
| **Aboriginal and Torres Strait Islander (n=978)** | | | | | | | | | | | | | | | |
| No diagnosis | 563 | 62.3  [55.0, 70.0] | 7.1  (<.001) |  | 499 | 55.1  [46.1, 63.3] | 1.9  (.062) |  | 475 | 54.8  [45.9, 63.1] | 1.4  (.176) | | 480 | | 54.1  [45.2, 63.2] |
| MI only | 49 | 4.2  [2.0, 7.6] | -2.1  (.034) |  | 51 | 4.6  [2.4, 7.9] | -2.0  (.050) |  | 48 | 4.4  [2.4, 7.8] | -0.1  (.953) | | 55 | | 4.7  [2.4, 7.9] |
| SUD only | 205 | 20.8  [17.4, 24.3] | -2.8  (.005) |  | 251 | 25.8  [20.6, 31.9] | 1.8  (.072) |  | 224 | 24.7  [20.3, 30.9] | -1.5  (.127) | | 234 | | 24.8  [20.4, 31.1] |
| Dual diagnosis | 161 | 12.6  [7.9, 16.8] | -4.2  (<.001) |  | 177 | 14.6  [10.5, 19.2] | -1.9  (.059) |  | 194 | 16.0  [10.6, 21.5] | -1.1  (.272) | | 209 | | 16.4  [10.4, 21.9] |
| Note. MDC = Major Diagnostic Category [61]. % = Bootstrapped prevalence estimates (10,000 repetitions) of subsample diagnosed using each data source and sample weighted by Australian prisoner population statistics, represented by percentage. CI = Bootstrapped bias-corrected confidence intervals. z-value = Z test comparing prevalence estimate from respective restricted data to unrestricted data from main paper. MI = Mental illness. SUD = Substance use disorder.  ^a^Inpatient and emergency department administrative data restricted to the five year’s preceding each participant’s baseline interview.  ^b^Dual diagnoses (as ascertained through inpatient and emergency department administrative data) defined as mental illness and substance use disorder diagnosed within the same 12-month period.  ^c^Individuals whose diagnoses relied solely on Major Diagnostic Categories removed from analyses; *n^Non-Indigenous^* = 1,651, *n^AboriginalTorresStraitIslander^* = 941.  ^d^Inpatient and emergency department administrative data unchanged from main analyses.  Non-Indigenous n = 1,667 unless otherwise stated. Aboriginal and Torres Strait Islander n = 978 unless otherwise stated. | | | | | | | | | | | | | | | |
